# Residential Selection across the Life Course: Adolescent Contextual and Individual Determinants of Neighborhood Disadvantage in Mid-Adulthood

**DOI:** 10.1371/journal.pone.0080241

**Published:** 2013-11-21

**Authors:** Per E. Gustafsson, Miguel San Sebastian, Urban Janlert, Töres Theorell, Hugo Westerlund, Anne Hammarström

**Affiliations:** 1 Department of Public Health and Clinical Medicine, Family Medicine, Umeå University, Umeå, Sweden; 2 Department of Public Health and Clinical Medicine, Epidemiology and Global Health, Umeå University, Umeå, Sweden; 3 Stress Research Institute, Stockholm University, Stockholm, Sweden; University of Stirling, United Kingdom

## Abstract

**Background:**

Numerous cross-sectional studies have examined neighborhood effects on health. Residential selection in adulthood has been stressed as an important cause of selection bias but has received little empirical attention, particularly its determinants from the earlier life course. The present study aims to examine whether neighborhood, family, school, health behaviors and health in adolescence are related to socioeconomic disadvantage of one's neighborhood of residence in adulthood.

**Methods:**

Based on the prospective Northern Swedish Cohort (analytical N = 971, 90.6% retention rate), information was collected at age 16 years concerning family circumstances, school adjustment, health behaviors and mental and physical health. Neighborhood register data was linked to the cohort and used to operationalize aggregated measures of neighborhood disadvantage (ND) at age 16 and 42. Data was analyzed with linear mixed models, with ND in adulthood regressed on adolescent predictors and neighborhood of residence in adolescence as the level-2 unit.

**Results:**

Neighborhood disadvantage in adulthood was clustered by neighborhood of residence in adolescence (ICC = 8.6%). The clustering was completely explained by ND in adolescence. Of the adolescent predictors, ND (b = .14 (95% credible interval = .07–.22)), final school marks (b = −.18 (−.26–−.10)), socioeconomic disadvantage (b = .07 (.01–.14)), and, with borderline significance, school peer problems (b = .07 (−.00–.13)), were independently related to adulthood ND in the final adjusted model. In sex-stratified analyses, the most important predictors were school marks (b = −.21 (−.32–−.09)) in women, and neighborhood of residence (ICC = 15.5%) and ND (b = .20 (.09–.31)) in men.

**Conclusions:**

These findings show that factors from adolescence – which also may impact on adult health – could influence the neighborhood context in which one will live in adulthood. This indicates that residential selection bias in neighborhood effects on health research may have its sources in early life.

## Introduction

Since the 1990s, a substantial body of empirical studies suggests a modest but fairly consistent association between socioeconomic characteristics of one's neighborhood environment, such as aggregated educational level or income, and physical and mental health, even after accounting for individual conditions [1], [2], [3], [4]. Less is known about circumstances that may influence the selection of people into certain neighborhoods, and to what degree the earlier life course matters for this residential selection. The present study employs a life course approach to the issue of residential selection, by examining whether circumstances in adolescence are related to disadvantage of one's neighborhood of residence in mid-adulthood.

The mere presence of residential segregation, e.g. by well-known social determinants of health such as social class, education and ethnicity, gives a simple illustration of the fact that people are not randomly distributed across neighborhoods. However, in the field of neighborhood effects on health, the neighborhood context is commonly treated as the causal starting point, with less attention paid to the antecedents of individuals' neighborhood context, or to how contextual factors and other social determinants of health interact over time. An understudied topic is thus residential selection, i.e., that individuals relocate to, or remain in, neighborhoods of certain characteristics, voluntarily or by financial or other restraints. Knowledge about residential selection is important for a broader understanding of how residential contexts and health interact, but it is hampered by the dominance of cross-sectional studies in the field [1], [2].

In stark contrast to the dominant focus on concurrent and recent circumstances in neighborhoods and health research, recent years have seen promising emerging conceptual [5], statistical [6], and empirical [7], [8], [9] efforts aiming to incorporate life course perspectives. Although consideration of the past life course so far has not been applied to the issue of residential selection, it is conceivable that residential selection in adulthood can operate along pathways stretching over long time periods and that the neighborhood context in which one ends up in adulthood thus has its roots in early life. For example, early mental health problems could impede an individual's entrance into the labor market, which could impact on future earnings and thereby limiting one's residential options to less affluent areas.

In addition to the conceptual and empirical merits of studying residential selection, the phenomenon has received considerable attention from a methodological point of departure. The focus has been on the tangible risk of selection bias and confounding which residential selection might introduce, and the fundamental challenges it therefore presents for drawing causal inference from studies on neighborhoods and health [10], [11], [12]. This has been deemed a key methodological problem of the entire field [10] and some authors even argue for eschewing observational studies [12]. When suitable data have been available, residential selection has commonly been addressed by considering duration of stay or moves during the last decade, e.g. by adjusting for change of area of residence [13], or by excluding individuals who have changed area [14]. Other approaches have been to measure accumulated exposure to neighborhood disadvantage (over 13 years) [15], or by investigating residential trajectories over even longer time periods in adulthood (20 years) [16]. Residential selection can introduce bias either by *direct selection*, whereby health status itself constitutes a residential selection factor, or by *indirect selection*, whereby risk factors for disease contribute to the residential selection [17]. Previous research has observed indirect selection effects, with those with more favorable socioeconomic circumstances (e.g. high education and income) tending to move to or remain in less deprived neighborhood over the course of 7–10 years [17], [18], but have found little indication of direct selection caused by mental health problems [18]. In the context of selection over the life course, numerous studies have also identified early life determinants for adult health, such as socioeconomic and family conditions [19], [20], [21], [22], school circumstances [23], [24], health behaviors [25], and health [26]. It is therefore, also from a methodological perspective, important to explore whether such factors from the early life course can influence residential selection in adulthood, as this could indicate risk for selection bias in studies on neighborhood effects on health in adulthood.

Therefore, the present prospective study aims to examine whether neighborhood, family, school, behavioral and health circumstances in adolescence are related to socioeconomic disadvantage of one's neighborhood of residence in adulthood.

## Methods

### Ethics statement

Ethical approval was granted by the Regional Ethical Review Board in Umeå. The retrieval and use of register data was also approved through a separate review of data safety and confidentiality by Statistics Sweden. Separate written informed consent was not requested by either committee, as the participants were regarded as giving written consent when completing the questionnaire at each data collection wave. All participants were clearly informed that participation in the study is voluntary and that they can decide to withdraw from participation at any time, without giving any explanation.

### Sample and procedures

The initial setting of the study is the Northern Swedish municipality of Luleå. Luleå City is the seat of the county administration and contains about two thirds of the population of the municipality (n = 42139/66869 in 1980 and n = 45467/72751 in 2005). Luleå City is a middle-sized industrial town, for which the metallurgic industries have been important, with rail connections to the Northern Swedish iron ore mines, a harbor connecting to the Baltic Sea, and steelworks. Since it opened in 1971, the university - the northernmost one in Sweden - has also been important for the city and the region. Luleå is comparable to Sweden as a whole with regard to e.g. labor market structure, housing, housing and socioeconomic status, but has had high levels of unemployment [27].

The Northern Swedish Cohort (NSC) is based on all school-leavers of the 9^th^ grade, the final grade of the Swedish compulsory school system, in the municipality of Luleå, in the year 1981, when the majority of participants were 16 years old. The eligible sample includes all who attended school as well as those who should have finished school this year but who had quit prematurely (n = 11). Individuals who went to special schools due to severe learning disability, visual impairment or hearing impairment were excluded, as well as one individual who was in long-term coma. There were 1083 eligible individuals, 1080 of whom participated in 1981. Four follow-up data collections waves (1983, 1986, 1995, 2008) have since then been conducted, see Hammarström and Janlert [28] for details of the NSC data and procedures. At the latest data collection in 2008, n = 1010 participated (94.3% of those 1071 individuals of the original sample still alive), 1001 of whom participated in the part of the study including retrieval of register data that is central for the present report. The Northern Swedish Cohort is conducted at Umeå University. The dataset is not freely available, and researchers interested in collaboration should get into contact with the Principal Investigator, Anne Hammarström.

Pertinent for the present report, in 1981 the participants completed comprehensive questionnaires about e.g. social conditions, health behaviors and health. Structured interviews about each participant were conducted with form teachers, information was retrieved from school records and blood pressure was measured.

In addition, each participant's neighborhood of residence at age 16 (1981) and 42 (2007) was linked to the cohort database. Neighborhood of residence was based on the Swedish population registry, and does therefore not include individuals living outside of Sweden or not having a registered address at all. Neighborhoods were defined as SAMS (Small-area market statistics) areas, a small-scale geographical division of Sweden by Statistics Sweden, with an average of about 1000 individuals living in each area. The areas are constructed as polygons with demarcations at roads and similar physically visible borders, with the intention to group buildings of similar type and appearance.

Register data covering all residents in all neighborhoods in which at least one cohort participant was residing in 1981 and 2007 was retrieved from Statistics Sweden. These data were used to construct aggregated measures of neighborhood disadvantage (ND) in adolescence and adulthood, see below. Participants were distributed across 72 neighborhoods in adolescence and across 374 neighborhoods in adulthood. The analytical sample of the present report comprised n = 971 individuals (467 women and 504 men), corresponding to 90.6% of the original cohort still alive (n = 1071) and 97.0% of those participating in adulthood (n = 1001). Due to non-response on particular items, the analytical sample varies between n = 926 and n = 971 in different models.

### Neighborhood disadvantage (ND) in adolescence and adulthood

Eight neighborhood indicators were aggregated at the neighborhood level at age 16 and 42 years. The selection was guided by previous research [29], [30], [31] and by the availability of register variables, with the aim to broadly cover socioeconomic conditions in a comparable manner at both measure points. See Table 1 for details of the operationalization and descriptive statistics of the individual indicators. Briefly, the indicators aggregated at the neighborhood level were: percentages of neighborhood residents with 1) *Low income*, 2) *High income* (reverse coded), 3) *Housing allowance*, 4) *Wealth* (reverse coded), 5) *Non-employment*, 6) *Single-parent household*, 7a) *Low occupational status* (only in 1981), 7b) *Low educational attainment* (only in 2007), 8a) *High occupational status* (reverse coded; only in 1981), and 8b) *High educational achievement* (reverse coded; only in 2007).

**Table 1:** Operationalization and descriptive statistics of neighborhood disadvantage indicators in 1981and 2007 measure points.

Label	Operationalization	Mean percentage (SD) across neighborhoods	
		1981	2007
1) Low income	Percentage of individuals living in a household *with an annual disposable household income per consumption unit* 1 in the household ≤10^th^ percentile of the Swedish population the corresponding year	10.4(4.8)	7.8(6.4)
2) High income	Percentage of individuals living in a household *with an annual disposable household income* per *consumption unit* 1 in the household ≥90^th^ percentile of the Swedish population the corresponding year (reverse coded)	9.9(6.5)	12.1(9.5)
3) Housing allowance	Percentage of individuals living in household receiving housing allowance	18.4(10.6)	5.4(5.9)
4) Wealth	Percentage of individuals paying any amount of wealth tax^2^ (reverse coded)	1.7(1.8)	3.2(3.5)
5) Non-employment	Percentage of adults (≥18 yrs) whose main income is from *unemployment*, *early retirement*, or *sickness benefits* or *compensation*; not counting income from retirement or employment.^3^	7.4(3.1)	6.7(3.3)
6) Single parent	Percentage of individuals living in single-parent households with one or more children	10.9(7.1)	7.8(3.7)
7a) Low occupational status	Percentage of individuals living in household with unskilled manual worker (SEI: 11–12) as the highest occupational level.	16.1(7.1)	N/A
7b) Low educational achievement	Percentage of individuals ≥25 yrs with only primary education, including primary education <9 years, and primary education 9–10) years	N/A	15.1(6.8)
8a) High occupational status	Percentage of individuals living in household with professionals or self-employed (SEI: 56–60) as the highest occupational level (reverse coded)	11.0(7.2)	N/A
8b) High educational achievement	Percentage of individuals ≥25 yrs with 2 or more years of tertiary education or PhD (reverse coded)	N/A	33.6(13.5)

1 *Disposable household income* is defined as the sum of household incomes from wages and salaries, entrepreneurial income and property income, plus current transfers received (incl e.g. earnings-related pensions and national pensions and other social security benefits, social assistance), minus transfers paid (including e.g. taxes, compulsory pension and unemployment insurance).Weighting for *consumptions units* is done by dividing the income by the sum of consumption unit weights: single-person household ( = 1.00); cohabitant couple ( = 1.51), additional adult ( = 0.60), first child 0–19 yrs ( = 0.52), second and additional children 0–19 yrs ( = 0.42).

2 Due to revocation of the Swedish wealth tax in 2007, the wealth tax for 2006 is used for the 2007 measurement.

3 Specifically, non-employment is based on an income variable categorizing all adults into one out of six mutual categories, based on the amount of income from different sources. The *basic amount* is calculated annually based on changes in the general price level, in accordance with the National Insurance Act (1962:381). The six categories, of which category 3, 4 and 5 are defined as non-employment, are: 1) employed ( = labor income more than 2 base amounts); 2) retired ( = not fulfilling the criteria for 1), and retirement pension >50% of the of the total income); 3) *early retirement* ( = not fulfilling the criteria for 1) or 2), and income from sickness compensation and activity compensation >50% of total income); 4) *unemployed* ( = not fulfilling the criteria for, 1) or 2) or 3), and income from unemployment benefits >50% of the total income); 5) *sick* ( = not fulfilling the criteria for 1) or 2) or 3) or 4), and income from sickness benefits >50% of the total income); 6) Other ( = not fulfilling the criteria for 1) or 2) or 3) or 4) or 5)).

All indicators were Z-transformed, then averaged across all indicators separately for 1981 and 2007, and finally the neighborhood scores were Z-transformed, yielding two continuous measures of *neighborhood disadvantage* (ND) in adolescence (age 16) and adulthood (age 42), respectively. Internal consistency (Cronbach's α) was estimated to be α = 0.89 (1981) and α = 0.89 (2007) at the neighborhood level, and α = 0.93 (1981) and α = 0.86 (2007) at the individual level.

### Family conditions in adolescence

Family conditions were operationalized through two variables from self-administered questionnaires, in accordance with our previous reports (see [19], [20], [21] for details). *Socioeconomic disadvantage* was based on parental occupation, which was classified into both parents with manual worker occupations ( = 1) and at least one parent with non-manual or self-employed occupations ( = 0). *Cumulative adversity* comprised on the sum of six dichotomized burdensome life conditions (range 0–6): residential crowding ( = not having own room), residential mobility ( = having moved more than three times during one's lifetime, corresponding to the 80th percentile), parental unemployment ( = either parent being unemployed), parental illness ( = either parent suffering from physical or mental illness, or having alcohol problems), parental separation or loss ( = parents being divorced, or either parent deceased) and low material standard of living ( = having less than four (80th percentile) items in the family's possession, from a list of eleven items, e.g. car and color TV).

### School adjustment in adolescence

School adjustment was operationalized through two variables [23]. *Finals school marks* of the 9^th^ grade were collected from school records, and categorized into decentiles, with those with no marks categorized as decentile 1. *School peer problems* comprised the sum (range 0–12,) of the two teacher-rated items ‘popularity-unpopularity among school peers’ and ‘tendency to isolation-extroversion’, each on a 6-level Likert scale.

### Health behaviors in adolescence

All health behaviors were measured by available items of the self-administered questionnaires [21], [23], [25]: *daily smoking* (yes = 1/no = 0); *physical activity* [six response options about frequency of physical activity during the last 12 months, ranging from ‘seldom/never’( = 1) to ‘each day’( = 6)]; *sedentary behavior* [proxied by an item about frequency of TV viewing with five response options, categorized into three levels ranging from ‘several shows a day’ ( = 1) to ‘one show every other day’ or less ( = 3)]; and *sugar consumption* [five response options about typical frequency of consumption of candy, sweets or pastries, ranging from ‘more seldom (than once each week)’( = 1) to ‘several times each day’( = 5)]. *Alcohol consumption* (estimated annual consumption of pure alcohol derived from questions about typical frequency and quantity of beverage consumption) displayed a substantially skewed distribution and was therefore categorized into quintiles (separately for women and men), collapsing quintiles 1 and 2 due to the high percentage of abstainers at the age of 16 years, yielding a four-level final variable.

### Mental and physical health in adolescence

Mental health variables were based on self-administered questionnaires. *Internalizing symptoms* were constructed from three items asking about worries/anxiousness, anxiety/panic, and sadness or feeling low, which were combined into a score (range 0–8). *Behavioral symptoms* comprised the sum (range 0–5) of the following five behavioral problems: truancy, driving car without license, vandalism, spending the night away from home without parents knowing, and been reported to the police. *Functional somatic symptoms* were operationalized as the sum (range 0–20) of the following ten symptoms: headache or migraine; other stomach ache; nausea; backache, hip pain or sciatica; fatigue; breathlessness; dizziness; overstrain; sleeplessness; and palpitations.

*Systolic and diastolic blood pressure* was measured in the student's right arm by trained medical personnel, with a standard sphygmomanometer, in the lying position after at least 10 minutes of resting. The mean of two readings was used. Weight and height were measured by school nurses as part of a compulsory health examination, and this information was retrieved from school health records to calculate *body mass index* (BMI, kg/m^2^).

### Data analysis

Prior to analysis, all variables were standardized to the grand mean/SD to aid the interpretation of the estimates.

The main analyses were directed at examining factors in adolescence as predictors of ND in adulthood, and were implemented using the user-written *runmlwin* command in Stata to fit multilevel models in the MLwiN software package v.2.23 [32]. Starting with restricted iterative generalized least squares estimation, we applied Markov Chain Monte Carlo (MCMC) methods, with a burn-in of 500, a chain length of 5,000 and a thinning interval of 1 [33]. Estimations with a burn-in of 5000, a chain length of 50,000 and a thinning interval of 10 did not change the results (data not shown). Fixed effects are reported as regression coefficients with 95% credible intervals (CrI), which can be interpreted in a similar way to confidence intervals [33]. Analyses comprises a series of random-intercept linear mixed models, all with individuals as the level-1 unit, neighborhood of residence (SAMS area) at age 16 as the level-2 unit, and ND at age 42 as the outcome. The analysis was done in three steps:

First, an empty model (Model 1) without any predictors was run, to estimate the degree of clustering (the intraclass coefficient, ICC) of the outcome by age 16 neighborhood of residence. Second, six independent models (Model 2a–e) with different sets of predictors were run, with the aim to estimate the fixed effects of age 16 predictors as well as the change in ICC. Third, the significant (p<.05) predictors from Model 2a–e were combined into a final adjusted model (Model 3).

In complementary analyses, the same three analytical steps were rerun stratified by sex, to explore whether the findings held true for women and men. As the sample sizes in these analyses were markedly lower (n = 467 women and 504 men, mean cluster size = 7.3–7.5), they are regarded as explorative.

Living in the same neighborhood in adolescence and adulthood was unrelated to ND in adulthood (t test, p = .542), and residential relocation was therefore excluded from the main analyses.

To examine if substantial multicollinearity was present, correlation matrices of fixed effects estimates were examined for all the mixed models. To explore whether the level of multicollinearity had any manifest impact on the estimates, all models with a correlation coefficient >.40 between two predictors were rerun twice. In the first rerun model, the first of the two collinear predictors was excluded while the second predictor was retained, and in the second rerun model, the second predictor was dropped and the first one was kept. If any of these two alternative models lead to different inferences compared to the original model, the collinear predictor with the weakest point estimate was dropped, while retaining the stronger predictor in the final model. The only instance where a predictor was dropped according to this procedure was alcohol consumption in the women-only analyses.

## Results

Table 2 shows descriptive statistics for all predictor variables by quintiles of ND at age 42. The cross tabulation of quintiles of ND at age 16 and 42 indicated a preponderance for individuals to live in similarly disadvantaged neighborhoods at age 42 as they did in adolescence. For example, individuals living in the least (quintile 1 or 2) or most (quintile 4 or 5) disadvantaged neighborhoods at age 16 were two to three times as likely to live in similarly disadvantaged neighborhoods at age 42 (24.4–34.0%) than to have moved to the extreme quintile at the opposite end of the spectrum (9.8–15.2%). Individuals of the middle quintile at age 16 were more evenly distributed across ND quintiles at age 42. Despite this measure of correspondence in neighborhood disadvantage over the life course, only 124 participants (12.7%) lived in the same neighborhood in both adolescence and adulthood.

**Table 2:** Selected descriptive statistics for all adolescent predictors by quintiles of ND at age 42.

Variables at age 16	Category and estimate shown	ND quintiles at age 42					P value
		Q1	Q2	Q3	Q4	Q5	
Neighborhood conditions							
Neighborhood disadvantage	Quintile 1 (row %)	34.0	20.9	20.4	9.4	15.2	<.001^a^
	Quintile 2 (row %)	24.4	24.4	22.3	16.2	12.7	
	Quintile 3 (row %)	17.9	25.0	17.9	21.7	17.4	
	Quintile 4 (row %)	9.8	16.7	20.1	30.9	22.5	
	Quintile 5 (row %)	11.3	18.5	17.4	21.0	31.8	
Family conditions							
Socioeconomic disadvantage	Manual worker parents (%)	22.9	35.0	36.9	40.2	53.6	<.001^b^
Cumulative adversity	Two or more adversities (%)	31.4	27.1	33.0	36.6.	49.2	<.001^c^
School adjustment							
Final school marks, decentiles	M(SD)	6.6(2.7)	5.6(2.8)	6.1(2.9)	5.2(2.8)	4.0(2.6)	<.001^c^
Peer problems	M(SD)	3.0(2.0)	3.5(2.2)	3.2(2.2)	3.7(2.2)	4.4(2.3)	<.001^c^
Health behaviors							
Alcohol consumption	Quintile 5 (%)	12.8	23.2	18.6	21.6	22.3	.046^b^
Smoking	Daily smoking (%)	19.7	25.7	24.5	24.7	37.0	<.001^b^
Physical activity	Daily physical activity (%)	13.3	7.9	10.7	8.2	6.2	<.001^c^
TV viewing	Several shows/day (%)	26.1	26.1	23.9	38.7	29.1	.064^b^
Sugar consumption	Several times a day (%)	2.7	3.5	2.1	3.1	4.7	.044^b^
Health							
Internalizing symptoms	M(SD)	1.1(1.4)	1(1.1)	1.3(1.6)	1.1(1.5)	1.2(1.3)	.360^c^
Behavioral symptoms	M(SD)	1.7(1.3)	2.1(1.4)	1.9(1.5)	2(1.5)	2.3(1.6)	<.001^c^
Functional somatic symptoms	M(SD)	3.3(2.5)	3.1(2.4)	3.5(2.4)	3.3(2.6)	3.5(2.7)	.398^c^
BMI	M(SD)	19.8(2.5)	19.8(2.5)	20.1(2.9)	20(2.8)	20.1(2.9)	.413^c^
Systolic BP	M(SD)	122.8(12.8)	121.7(13.4)	120.6(13.4)	121.9(13.5)	120.9(12.9)	.277^c^
Diastolic BP	M(SD)	68.8(10.3)	68.9(10.2)	69.3(11.4)	68.2(11.7)	69.8(12.1)	.739^c^

Note that for ordinal variables except quintiles of ND at age 16, descriptives are shown only for a single (collapsed) category, as indicated in the table. Bivariate associations were however estimated using the full range of each variable.

a p value from χ^2^.

b p value from Mantel-Haenszel test.

c p value from Spearman's ρ.

With respect to the individual-level predictors in Table 2, individuals who lived in more disadvantaged neighborhoods in adulthood came from more disadvantaged families, had been exposed to more adversity, and had lower school marks and more problems with school peers. With regard to health behaviors, they smoked more and engaged less in physical activity in adolescence, and tended to eat more sweets, drink more alcohol and watch more television. They also reported more behavioral symptoms, but did not differ in any other health measures.

See Table 3 for a summary of linear mixed models where ND in adulthood is regressed on factors in adolescence in the total sample. The empty model (Table 3, Model 1) estimated that 8.6% of the variance in ND in adulthood was explained by area of residence in adolescence. Adolescent ND (Model 2a) almost completely explained this clustering, with ICC dropping to 1.1%. In models with individual-level predictors only (Model 2b–e), family conditions explained the largest portion of neighborhood clustering (Model 2b, ICC = 4.3%), followed by school adjustment (Model 2c, ICC = 6.1%), whereas adolescent health behaviors (Model 2d ICC = 7.9%) and adolescent health (Model 2e, ICC = 7.7%) did not seem to explain the clustering to a substantial degree.

**Table 3:** Summary of linear mixed models in the total sample: age 16 predictors of neighborhood disadvantage at age 42, with neighborhood of residence at age 16 as the level-2 unit.

Adolescent predictors	Model 1	Model 2a	Model 2b	Model 2c	Model 2d	Model 2e	Model3
Neighborhood conditions							
Neighborhood disadvantage		**.25 (.18–.31)**					**.14 (.07–.22)**
Family conditions							
Socioeconomic disadvantage			**.14 (.07–.20)**				**.07 (.01–.14)**
Cumulative adversity			**.11 (.04–.17)**				.03 (−.03–.09)
School adjustment							
Final school marks				**−.23 (−.29–−.16)**			**−.18 (−.26–−.10)**
Peer problems				**.08 (.01–.15)**			.07 (−.00–.13)
Health behaviors							
Alcohol consumption					−.05 (−.13–.02)		
Smoking					**.09 (.02–.16)**		.01 (−.06–.08)
Physical activity					**−.11 (−.18–−.04)**		−.03 (−.10–.03)
TV viewing					**.07 (.01–.13)**		.02 (−.03–.09)
Sugar consumption					.06 (−.01–.12)		
Health							
Internalizing symptoms						−.01 (−.08–.07)	
Behavioral symptoms						**−.08 (.01–.14)**	−.03 (−.10–.04)
Functional somatic symptoms						.03 (−.04–.11)	
BMI						.02 (−.04–.09)	
Systolic BP						−.02 (−.09–.04)	
Diastolic BP						.02 (−.05–.08)	
Random effects							
Individual-level variance	.92	.92	.91	.85	.88	.93	.86
Neighborhood-level variance	.08	.01	.04	.06	.08	.08	.01
ICC (%)	8.6	1.1	4.3	6.1	7.9	7.7	1.6
Bayesian DIC	2710	2695	2667	2595	2641	2628	2512
Model descriptives							
N level 2 units	72	72	72	72	72	71	72
N individuals	971	971	961	955	957	936	927
Mean cluster size	13.5	13.5	13.3	13.3	13.3	13.2	12.9

Numbers are fixed effects estimates (credible intervals) unless otherwise noted. All variables are standardized.

Concerning fixed effects, ND in adolescence was significantly related to the corresponding measure at age 42, with one SD higher in ND in adolescence corresponding to a 0.25 (95% CrI = .18–.31) SD higher ND at age 42 (Model 2a). Of the individual-level predictors, socioeconomic disadvantage, cumulative adversity (Model 2b), final school marks, peer problems (Model 2c), smoking, physical activity (Model 2d) and behavioral problems (Model 2e) were found to relate to ND in adulthood. Combining the significant predictors from Model 2a–e in Model 3 showed that the most important independent predictors were final school marks (b = −.18 (−.26–.−.10)) and ND (b = .14 (.07–.22)), followed by socioeconomic disadvantage (b = .07 (.01–.14)), with school peer problems reaching borderline significance (b = .07 (−.00–.13)).

In complementary analyses, all models were rerun stratified by sex (see Table 4 for results in women and Table 5 for results in men). The empty model (Model 1) indicated that neighborhood of residence in adolescence explained a substantial part of adulthood ND in men (ICC = 15.5%), while the degree of clustering was numerically less impressive in women (ICC = 5.0%). Consistent with the results in the total sample, however, the clustering was largely explained by adolescent ND, which also was strongly related to adult ND, in both women and men (Model 2a). The importance of family conditions (Model 2b) and school adjustment (Model 2c) in women and men was largely comparable to findings in the total sample, with the exceptions that in women, the peer problem estimate was weak and decidedly non-significant, and that family conditions jointly explained a considerable amount of the clustering by neighborhood (Model 2b, ICC = 1.4%). With regard to health behaviors (Model 2d) the only significant fixed effects were smoking in women and physical activity in men, with TV viewing reaching borderline significance in men. Of the health measures (Model 2e), functional somatic symptoms was significant in women while in men, only behavioral symptoms reached borderline significance. In the combined model (Model 3) in men, ND was the strongest predictor (b = .20 (95% CrI = .09–.31) followed by school marks (b = −.14 (−.24–−.03)) and peer problems (b = .10 (.01–.19), with none of the other predictors remaining significant. In women, school marks was the clearly strongest predictor (b = −.21 (−.32–−.09)), followed by ND (b = .11 (.00–.22)) and the borderline significant functional somatic symptoms (b = .09 (−.00–.19).

**Table 4:** Summary of linear mixed models in women: age 16 predictors of neighborhood disadvantage at age 42, with neighborhood of residence at age 16 as the level-2 unit.

Adolescent predictors	Model 1	Model 2a	Model 2b	Model 2c	Model 2d	Model 2e	Model3
Neighborhood conditions							
Neighborhood disadvantage		**.21 (.11–.31)**					**.11 (.00–.22)**
Family conditions							
Socioeconomic disadvantage			**.15 (.05–.25)**				.08 (−.02–.18)
Cumulative adversity			**.16 (.07–.25)**				.07 (−.02–.17)
School adjustment							
Final school marks				**−.27 (−.36–−.17)**			**−.21 (−.32–−.09)**
Peer problems				.03 (−.07–.13)			
Health behaviors							
Alcohol consumption						—a	
Smoking					**.11 (.01–.20)**		−.00 (−.10–.10)
Physical activity					−.05 (−.16–.06)		
TV viewing					.07 (−.03–.17)		
Sugar consumption					.06 (−.03–.15)		
Health							
Internalizing symptoms						−.05 (−.14–.05)	
Externalizing symptoms						−.06 (−.06–.17)	
Functional somatic symptoms						**.15 (.03–.26)**	.09 (−.00–.19)
BMI						.00 (−.09–.10)	
Systolic BP						−.00 (−.11–.11)	
Diastolic BP						−.00 (−.10–.09)	
Random effects							
Individual-level variance	1.03	1.03	1.02	.95	1.02	1.04	.96
Neighborhood-level variance	.05	.02	.01	.03	.03	.05	.02
ICC (%)	5.0	1.6	1.4	3.2	3.2	4.6	2.1
Bayesian DIC	1357	1345	1324	1293	1337	1325	1291
Model descriptives							
N level 2 units	62	62	62	62	62	62	62
N individuals	467	467	461	459	461	454	456
Mean cluster size	7.5	7.5	7.4	7.4	7.4	7.3	7.4

a The predictor was dropped from the model due to multicollinearity.

Numbers are fixed effects estimates (credible intervals) unless otherwise noted. All variables are standardized.

**Table 5:** Summary of linear mixed models in men: age 16 predictors of neighborhood disadvantage at age 42, with neighborhood of residence at age 16 as the level-2 unit.

Adolescent predictors	Model 1	Model 2a	Model 2b	Model 2c	Model 2d	Model 2e	Model3
Neighborhood conditions							
Neighborhood disadvantage		**.29 (.19–.38)**					**.20 (.09–.31)**
Family conditions							
Socioeconomic disadvantage			**.14 (.05–.23)**				.06 (−.02–.15)
Cumulative adversity			.06 (−.02–.15)				
School adjustment							
Final school marks				**−.18 (−.28–−.10)**			**−.14 (−.24–−.03)**
Peer problems				**.13 (.05–.22)**			**.10 (.01–.19)**
Health behaviors							
Alcohol consumption					−.03 (−.12–.06)		
Smoking					.03 (−.07–.14)		
Physical activity					**−.15 (−.23–−.06)**		−.07 (−.15–.02)
TV viewing					.08 (−.01–.16)		
Sugar consumption					.04 (−.04–.12)		
Health							
Internalizing symptoms						.04 (−.11–.18)	
Behavioral symptoms						**.09 (.00–.18)**	.00 (−.09–.10)
Functional somatic symptoms						−.07 (−.17–.02)	
BMI						.04 (−.05–.12)	
Systolic BP						−.04 (−.12–.05)	
Diastolic BP						.05 (−.04–.13)	
Random effects							
Individual-level variance	.80	.82	.79	.77	.76	.81	.78
Neighborhood-level variance	.15	.03	.11	.08	.12	.13	.03
ICC (%)	15.5	4.1	12.0	9.6	14.0	13.6	3.1
Bayesian DIC	1350	1345	1334	1309	1309	1306	1265
Model descriptives							
N level 2 units	67	67	67	67	67	66	67
N individuals	504	504	500	496	496	482	479
Mean cluster size	7.5	7.5	7.5	7.4	7.4	7.3	7.1

Numbers are fixed effects estimates (credible intervals) unless otherwise noted. All variables are standardized.

## Discussion

The present study suggests that adolescent neighborhood, family and school circumstances, but not health nor health behaviors, are independently predictive of the socioeconomic character of one's neighborhood of residence in mid-adulthood. Overall, our findings are thus consistent with indirect residential selection effects, i.e. that risk factors for poor health relate to residential selection, operating over the life course.

The empty model clustering of adult ND by adolescent neighborhood of residence was estimated at 8.6%. This could be considered a comparatively low level of clustering, particularly when considering that both the cluster unit and the outcome are derived from neighborhood of residence. At the same time, a period of 26 years had passed between the time points, during which a multitude of exposures could affect one's choice, and ability to choose, area of residence in adulthood. To the degree that such exposures are unrelated to early life residential location, they would attenuate the clustering. As such, the estimated clustering could be viewed as quite substantial. Nevertheless, the degree of clustering sets the limit for how relevant between-neighborhood differences are for the individual variation of the outcome [34], and it is important to note that the largest portion of ND variability in adulthood seems to be unrelated to one's neighborhood of residence in adolescence.

Our findings suggest that the clustering was largely explained by ND in adolescence, which together with the robust association between ND in adolescence and adulthood suggests a degree of continuity in one's residential context across the life course. This was also illustrated by the descriptive cross tabulation of ND quintiles in adolescence and adulthood, in which particularly individuals in with high or low ND in adolescence tended to live in similarly disadvantaged neighborhoods in adulthood. Interestingly, as only a minority of participants (12.7%) lived in the same neighborhood in adolescence and adulthood, the continuity of neighborhood disadvantage over the life course does not seem to be explained by lack of residential mobility, i.e. that people simply remain in the same neighborhoods across the life course. The results also seem to suggest that the individual-level variables considered were not sufficient to completely explain the association of ND in adolescence and adulthood. It is possible that the residential continuity is partly explained by residential preferences established during upbringing, or constraining conditions not considered in the analysis, such as income.

Family conditions were the set of individual-level predictors most clearly explaining the clustering of adult ND, indicating a compositional effect, which seems reasonable considering that these factors are intimately tied to one's residential environment. As we have reported previously [35], about 40% of the cohort remained in either blue-collar or white-collar socioeconomic categories from adolescence to adulthood. The importance of one's class of origin could therefore partly be explained by social immobility, and the opportunities or constraints socioeconomic conditions in adulthood imply for one's residential options.

One notable finding is that school adjustment factors, particularly academic achievement and to a lesser degree functioning among school peers, were strong predictors of one's neighborhood context in adulthood. Together with our previous reports of how these factors relate to metabolic health in adulthood [23], [36], the finding illustrates the wide-ranging and enduring impact of successful or unsuccessful adjustment to the institutional demands placed on young people. It is conceivable that early academic success together with one's socioeconomic background sets about a chain of events over the life course, such as later educational achievement, job opportunities [36] and earnings, and thereby influences one's possibilities to choose area of residence. The importance of family and school circumstances for one's adult neighborhood context is thus consistent with, and could represent the antecedent of, the more short-term indirect residential selection effect by socioeconomic factors demonstrated in previous research [17], [18].

Physical activity has previously been highlighted as a potential residential selection factor [37], [38]. Our results suggest that this selection effect, at least as indicated in adolescence, might largely be explained by social factors such as earlier neighborhood, family and school conditions.

We found no support for direct selection effects of the health variables examined in the total sample. Although behavioral symptoms, like physical activity, were significantly related to ND in adulthood in unadjusted and moderately adjusted analyses, which is indicative of a direct selection effect, it was attenuated below significance consideration of other social circumstances. This also points to the complexity of examining selection effects, where an apparent candidate itself may be confounded by other factors.

The sample sizes of the sex-stratified analyses were small, so these results should be viewed as explorative and interpreted cautiously. Despite that the degree of neighborhood disadvantage was similar in women and men, the relative importance of the predictors displayed different patterns. For men, neighborhood of origin seemed to play a large role in explaining adult neighborhood disadvantage, but considerably less so for women. In women, final school marks instead emerged as the single most important factor, seemingly superseding the contextual influences. This could possibly be explained by the protective effects of early academic success against gendered psychosocial risks, such as early parenthood [39], [40], which may have enduring consequences for particularly women's future possibilities to educational, occupational [41], and potentially also residential, prospects. Another finding was that functional somatic symptoms tended to be independently predictive of adult neighborhood disadvantage in women only, which suggests a possible direct selection effect. Future studies should further examine to which degree residential selection over the life course unfold by gendered patterns.

The findings of this study also illustrate the serious concerns about causal inference in neighborhood and health studies discussed by Oakes [12] and others [10], [42]. From a counterfactual causal framework [12], our results can be interpreted as a demonstration of the situation that (groups of) individuals living in neighborhoods of different characteristics, by virtue of disparate life course exposures, are not exchangeable with their (unobservable) counterfactual comparison. As a corollary, under these conditions estimation of causal neighborhood effects on health would be invalid. Moreover, and in addition to the inherent limitations of adjusting for selection effects by means of multiple variable analyses (e.g. regression models) [12], two additional features of our results might be particularly relevant for neighborhood and health research. First, several of the selection factors could potentially impact on adult health, and as such could introduce bias in cross-sectional neighborhood and health studies. Second, while some of these factors possibly may act through easily measurable adult factors, e.g. through social chains of risk as described above, some early life factors have the potential to act on later health independently of adult circumstances, e.g. enduring health impact of early life social class, independently of later circumstances (see e.g. [43], [44], [45], [46]). In the former case, one could argue that adjustment for a causally proximal factor, e.g. adult social class, would mitigate the confounding effect of antecedent selection factors. In the latter and more worrisome case, adjustment for concurrent or recent social circumstances would not be sufficient to take into account the bias introduced by early life selection factors, as there might not be any easily measureable mediator that captures both the selection effect and the health effect. A recommendation for future neighborhood and health studies is therefore, whenever possible, to collect information on social background factors during early life. Parental occupation or educational attainment are examples of variables that could be feasible to record even for cross-sectional studies, as these could potentially be reported retrospectively or retrieved from population registries and censuses.

Although the present study did not specifically focus on migration and residential mobility, some particular issues are worth commenting upon. As much as 87.3% of participants lived in a different neighborhood at age 42 compared to at age 16. Moreover, this percentage does not take into consideration any additional moves between these two time points, for example, people moving out of their parents' house in young adulthood and then moving back at a later stage in life, due to inheriting the dwelling from aged or deceased parents. Taking into consideration participants' neighborhood of residence at age 21 and 30 years (data not included in the present report) shows that only 45 individuals (4.7%) lived in the same neighborhood at age 16, 21, 30 and 42. Although there was no difference in adult ND between those who had moved versus those who had stayed in the same neighborhoods, this suggests a high degree of social mobility over the life course. Relatedly, the moderately strong correspondence between ND at age 16 and 42 suggests that early life neighborhood context is important for later residential context, but not to the degree that the measures should be considered equivalent, or that timing of measurement could be considered irrelevant. Incorporating longitudinal measurement of neighborhood of residence, such as accumulation [15] or trajectories [16] of neighborhood disadvantage, thus appear to be more attractive options than recording residential area at one time point.

### Methodological considerations

The strengths of this study include the prospective design, high retention rate, a sample representative of the corresponding age cohort in Sweden, and the multiple data sources.

The sample is based on a set of individuals of a specific age living in a specific region at a specific time point, and it is possible that cohort effects influence the patterns of residential mobility and the relative importance of determinants estimated in this cohort. For example, although the cohort was comparable to the Swedish population with respect to a number of background variables [27], Northern Sweden is a region with historically higher unemployment rates compared to Sweden as a whole, which possibly could force people to move to pursue job opportunities to a greater degree than in other parts of Sweden. Moreover, demographic changes in Sweden since the initiation of the study, for example with regard to immigration and fraction of young adults studying at university, could also have importance for residential mobility patterns. Such influences would limit the external validity of the results, and should be taken into consideration when generalizing the findings.

Another crucial methodological issue is the validity of the geographical boundaries of neighborhood, a topic that has received increased attention in area effects on health research in recent years [13], [15], [42]. The use of administrative boundaries, such as the SAMS, has been criticized for not necessarily being valid demarcations of the ‘collective bodies’ which neighborhoods are assumed to represent in area effects on health research [42]. For example, one study showed that the clustering of all-cause mortality by the administrative boundaries of census tract or municipality were small compared to the household level [13], and the ICC for ischemic heart disease by SAMS areas has in previous studies been estimated at a mere 1.5–2.5% [15], [47]. As such, our findings are relevant for area effects on health research only to the degree that SAMS areas actually represent valid boundaries for the clustering of health. In addition, the geographical boundaries of the SAMS areas were defined in 1994 and are comparable across the years. Although this makes the areas suitable for longitudinal comparisons, the fixed nature of the boundaries also means that changes in the neighborhoods, such as real estate changes, are not considered. This could mean that the validity of the boundaries may differ between the years. Moreover, the patterns and determinants of residential mobility would be expected to depend on the size of geographical unit. For example, although the different age of participants and time period considered limit comparisons to the present study, Merlo et al. [13] report that, in an Andalusian sample of middle-aged to older adults, only 3 to 10% (depending on educational level) changed municipality over a 10-year period, compared to the 87.3% changing SAMS area over the 26-year period in the present study.

Although mutual adjustment of predictors is necessary to take confounding into account, the estimated independent contribution of a predictor in multiple-variable analysis is substantially influenced by the metric properties of the variable, which might lead to misestimation of the true independent effects, such as the selection of the predictors to the final model. Even though overt collinearity was handled by omission of variables, complex causal relations between predictors might also lead to over-adjustment.

Although descriptive analyses (Table 2) suggested that the correspondence between neighborhood disadvantage in adolescence and adulthood were higher for more extreme disadvantage in adolescence, extending the mixed models to include a random slope for ND in adolescence did not change the estimates (data not shown).

The statistical impact of small sample sizes in multilevel models is a matter of contention. Very small cluster sizes (n = 1–5) in combination with few level-2 units (n<100) have been shown to increase Type 2 error rate and contribute to upward bias of the ICC estimate [48]. However, clusters sizes of about n = 5 have been shown to yield valid estimates, with fixed effect estimated more robustly than random effects estimates [49]. Small level-2 sample size has also been shown to impact more on level-2 than level-1 fixed estimates [50]. In conclusion, the small sample sizes of particularly the sex-stratified analyses may bias the random and level-2 fixed effects, and these estimates should therefore be interpreted with caution.

## Conclusions

This study suggests that one's neighborhood context in adulthood is partially rooted in neighborhood, family and school circumstances during the early life course. These results highlight the complex relationships between contextual and individual social determinants of health over the life course, and the potential confounding role early life factors can play in studies on neighborhood effects on health in adulthood. Future studies should further examine how residential selection unfolds over the life course, and how this should be approached by studies on neighborhood effects on health. Moreover, to mitigate the potential confounding effect of early life selection factors, we would recommend studies about neighborhood effects on health to measure area of residence and disadvantage longitudinally or at least to retrospectively collect information on circumstances during upbringing, such as parental occupation.
